# BAR-encapsulated nanoparticles for the inhibition and disruption of *Porphyromonas gingivalis*–*Streptococcus gordonii* biofilms

**DOI:** 10.1186/s12951-018-0396-4

**Published:** 2018-09-15

**Authors:** Mohamed Y. Mahmoud, Donald R. Demuth, Jill M. Steinbach-Rankins

**Affiliations:** 1 Department of Oral Immunology and Infectious Diseases, University of Louisville School of Dentistry, 501 S. Preston St, Louisville, KY 40202 USA; 2 Department of Bioengineering, University of Louisville Speed School of Engineering, 505 S. Hancock St., Room 623, Louisville, KY 40202 USA; 3 Department of Microbiology and Immunology, University of Louisville School of Medicine, Louisville, KY 40202 USA; 4 Department of Pharmacology and Toxicology, University of Louisville School of Medicine, Louisville, KY 40202 USA; 5 Center for Predictive Medicine, University of Louisville, 505 S. Hancock St, Louisville, KY 40202 USA

**Keywords:** Polymer nanoparticle, Poly(lactic-*co*-glycolic acid), Peptide delivery, Drug delivery, *Porphyromonas gingivalis*, *Streptococcus gordonii*, Periodontal disease, Oral biofilm

## Abstract

**Background:**

*Porphyromonas gingivalis* adherence to oral streptococci is a key point in the pathogenesis of periodontal diseases (Honda in Cell Host Microbe 10:423–425, 2011). Previous work in our groups has shown that a region of the streptococcal antigen denoted BAR (SspB Adherence Region) inhibits *P. gingivalis/S. gordonii* interaction and biofilm formation both in vitro and in a mouse model of periodontitis (Daep et al. in Infect Immun 74:5756–5762, 2006; Daep et al. in Infect immun 76:3273–3280, 2008; Daep et al. in Infect Immun 79:67–74, 2011). However, high localized concentration and prolonged exposure are needed for BAR to be an effective therapeutic in the oral cavity.

**Methods:**

To address these challenges, we fabricated poly(lactic-*co*-glycolic acid) (PLGA) and methoxy-polyethylene glycol PLGA (mPEG-PLGA) nanoparticles (NPs) that encapsulate BAR peptide, and assessed the potency of BAR-encapsulated NPs to inhibit and disrupt in vitro two-species biofilms. In addition, the kinetics of BAR-encapsulated NPs were compared after different durations of exposure in a two-species biofilm model, against previously evaluated BAR-modified NPs and free BAR.

**Results:**

BAR-encapsulated PLGA and mPEG-PLGA NPs potently inhibited biofilm formation (IC50 = 0.7 μM) and also disrupted established biofilms (IC50 = 1.3 μM) in a dose-dependent manner. In addition, BAR released during the first 2 h of administration potently inhibits biofilm formation, while a longer duration of 3 h is required to disrupt pre-existing biofilms.

**Conclusions:**

These results suggest that BAR-encapsulated NPs provide a potent platform to inhibit (prevent) and disrupt (treat) *P. gingivalis/S. gordonii* biofilms, relative to free BAR.

**Electronic supplementary material:**

The online version of this article (10.1186/s12951-018-0396-4) contains supplementary material, which is available to authorized users.

## Background

Periodontal disease is a group of chronic inflammatory diseases commonly caused by *Porphyromonas gingivalis*, *Tannerella forsythia*, and *Treponema denticola*. Together these pathogens are known as the “red complex” [1]. The progression of periodontal disease can cause tissue destruction and tooth loss, and if left untreated can contribute to systemic conditions of increased cancer risk, cardiovascular disease, diabetes, rheumatoid arthritis, pulmonary disease, and obesity [2, 3].

Current periodontal treatments aim to reduce bacterial plaque formation in the oral cavity using primarily physical and chemical (antibiotic) methods [4, 5]. However, current antibiotic treatment strategies exhibit non-specific activity, affecting beneficial organisms also present in the oral microbiome. Additional potential risks include the development of anti-bacterial resistant species, emergence of fungal opportunistic infections or *Pseudomonas* infection, and allergic reactions. Last, most current antibiotics have difficulty penetrating periodontal biofilms, and must be frequently administered, due to their transient activity in the oral cavity [6–8].

*Porphyromonas gingivalis* has been found to be associated with chronic periodontitis in 88% of sub-gingival plaque samples [9]. Moreover, *P. gingivalis* and *S. gordonii* association enhances the disruption of host–microbe homeostasis and induces population changes in the subgingival biofilm, driving inflammatory periodontal diseases [10–12]. Previous work in our group has shown that *P. gingivalis* adherence to *streptococci* is driven by the interaction of the minor fimbrial antigen (Mfa) of *P. gingivalis* and the streptococcal antigen I/II (AgI/II) [13, 14]. From these studies, a peptide (designated BAR), was developed that potently inhibits *P. gingivalis*/*S. gordonii* adherence in vitro and reduces *P. gingivalis* virulence in a mouse model of periodontitis [15–17]. While efficacious, one of the challenges to free BAR administration is that it provides relatively transient inhibition of *P. gingivalis* in the oral cavity. Moreover, to treat established biofilms, relative to initial biofilm formation, higher concentrations of BAR are required.

Polymeric delivery vehicles provide one option to address these challenges, by offering prolonged and targeted delivery of active agents. In particular, for application to the oral cavity, polymeric nanoparticles (NPs) are easy to fabricate and produce stable formulations. From a delivery perspective, polymeric NPs may offer rapid degradation in the acidic environment of the oral cavity, while providing mucoadhesive properties due to the electrostatic interactions between NPs and gingival epithelium [18–20]. Furthermore, for more labile molecules like biologics, polymers have the potential to protect the functionality of the active agent and provide tunable release and prolonged delivery, while enabling localization of the active agent to target sites [19, 21]. In addition polymeric NPs may offer a safer and more biocompatible delivery method, relative to currently applied metallic NPs that exhibit broad antimicrobial effect [22, 23].

Previous work in our groups has demonstrated that NPs surface-modified with BAR peptide more potently inhibit *P. gingivalis* adherence to *S. gordonii*, relative to an equimolar administration of free BAR peptide in vitro [24]. This increased potency was attributed to a higher localized dose of BAR, facilitating multivalent interactions with *P. gingivalis.* While surface-modified NPs provide targeting efficacy, a method of delivering high concentrations of BAR for prolonged duration has not been investigated. In this study, we sought to develop a formulation that encapsulates and prolongs the delivery of BAR, for durations relevant to oral delivery. BAR-encapsulated PLGA NPs were characterized and evaluated in two-species biofilm inhibition and disruption models. In addition, the kinetics of BAR-encapsulated, relative to BAR surface-modified NPs were assessed in a two-species model.

## Methods

### Peptide synthesis

BAR peptide is comprised of residues 1167 to 1193 of the SspB (Antigen I/II) protein sequence of *S. gordonii* (NH_2_-LEAAPKKVQDLLKKANITVKGAFQLFS-COOH) [16]. To enable peptide quantification and detection, the epsilon amine of the underlined lysine residue of BAR was covalently reacted with 6-carboxyfluorescein to produce fluorescent BAR (F-BAR). Both unlabeled and labeled peptides were synthesized by BioSynthesis, Inc. (Lewisville, TX) and obtained with greater than 90% purity.

### BAR-encapsulated and BAR surface-modified nanoparticle synthesis

BAR and F-BAR encapsulated poly(lactic-*co*-glycolic acid) PLGA and methoxy-polyethylene glycol (mPEG-PLGA) NPs were synthesized using a double emulsion technique [25, 26]. Briefly, BAR was encapsulated in PLGA carboxyl-terminated polymer (0.55–0.75 dL/g; LACTEL^®^; DURECT Corporation, Cupertino, CA, USA) or mPEG-PLGA (Mw ~ 5000:55,000 Da; PolySciTech^®^; Akina, Inc., IN, USA). One hundred milligrams of PLGA or mPEG-PLGA was dissolved in 2 mL methylene chloride (DCM) overnight. The next day, BAR was dissolved in 200 μL Tris EDTA (TE) buffer at a concentration of 43 μg BAR/mg PLGA. The resulting PLGA/DCM solution was vortexed while adding 200 μL of BAR peptide solution dropwise, and the mixture was ultrasonicated. Next, 2 mL of the PLGA/DCM/BAR solution was added dropwise to 2 mL of 5% (w/v) polyvinyl alcohol (PVA) while vortexing and was subsequently sonicated. The NP solution was added to 50 mL of 0.3% PVA for 3 h to evaporate residual DCM. After evaporation, the NP solution was centrifuged at 13,000 rpm at 4 °C and washed with distilled water twice. F-BAR encapsulated NPs were synthesized similarly, but were protected from light to avoid photobleaching.

BAR surface-modified NPs were synthesized similarly as above using a previously described double emulsion technique [26–29]. Briefly, the 5% (w/v) polyvinyl alcohol (PVA) solution was mixed with 2 mL of 5 mg/mL avidin-palmitate and the 2 mL PLGA/DCM solution was added dropwise to 4 mL PVA/avidin-palmitate while vortexing. After the first wash, the supernatant was discarded and the pelleted NPs were resuspended in 10 mL PBS for 30 min on a benchtop rotator, with biotinylated BAR peptide at a molar ratio of 3:1 BAR:avidin (18.5 nmol/mg) in PBS. After conjugation, the NPs were washed two times with distilled water by centrifugation at 13,000 rpm at 4 °C. After washing, both BAR-encapsulated and BAR surface-modified NPs, were suspended in 5 mL of distilled water, frozen at − 80 °C, and lyophilized.

### NP characterization: NP morphology, size, BAR loading, controlled release

Unhydrated NP morphology, diameter, and size distribution were determined by analyzing scanning electron microscopy (SEM) images with NIH ImageJ software (version 1.5a, imageJ.nih.gov). Dynamic light scattering and zeta potential analyses were performed on hydrated NPs to determine the hydrodynamic diameter and surface charge (Malvern, Malvern, UK). To determine BAR loading and encapsulation efficiency (EE), NPs were dissolved in dimethyl sulfoxide (DMSO). The quantity of extracted F-BAR was determined by measuring fluorescence (488/518 nm excitation/emission). For BAR-encapsulated NPs, in vitro release was measured by gentle agitation of NPs in phosphate buffered saline (PBS, pH 7.4) at 37 °C. At fixed time points (1, 2, 4, 8, 24, 48 h), samples were collected and the amount of BAR released from the NPs was quantified as described above.

### Growth of bacterial strains

*Porphyromonas gingivalis* ATCC 33277 was grown in Trypticase soy broth (Difco Laboratories Inc., Livonia, MI, USA) supplemented with 0.5% (w/v) yeast extract, 1 μg/mL menadione, and 5 μg/mL hemin. The medium was reduced for 24 h under anaerobic conditions (10% CO_2_, 10% H_2_, and 80% N_2_) and *P. gingivalis* was subsequently inoculated and grown anaerobically for 48 h at 37 °C. *S. gordonii* DL-1 was cultured aerobically without shaking in brain–heart infusion broth (Difco Laboratories Inc.) supplemented with 1% yeast extract for 16 h at 37 °C.

### Biofilm inhibition assay

To assess the effectiveness of BAR-encapsulated NPs to prevent the interaction of *P. gingivalis* with *S. gordonii*, *S. gordonii* was harvested from culture and labeled with 20 μL of 5 mg/mL hexidium iodide for 15 min at room temperature. Following incubation, cells were centrifuged to remove unbound fluorescent dye. Subsequently, the bacterial concentration was measured by the O.D. at 600 nm from 20-fold diluted cultures of *S. gordonii*. The optical density of *S. gordonii* cells was adjusted to 0.8 (1 × 10^9^ CFU/mL) to obtain uniformity between cell counts in each well. After adjusting the optical density, 1 mL of *S. gordonii* cells was added to each well of 12-well culture plates containing a sterilized micro-coverslip. The cell culture plates were wrapped in aluminum foil to protect the labeled cells from light and placed on a rocker platform in the anaerobic chamber for 24 h.

*Porphyromonas gingivalis* cultures were optimized using a similar approach, utilizing a different fluorescent label (20 μL of 4 mg/mL carboxyfluorescein–succinylester). *P. gingivalis* was incubated with the fluorescent dye for 30 min on a rocker platform and protected from light. The same procedures were followed as performed with *S. gordonii* to determine cell concentration, with slight adaptations. The optical density of *P. gingivalis* was adjusted from 0.8 to 0.4 O.D. (5 × 10^7^ CFU/mL) by diluting *P. gingivalis* cultures with an equal volume of BAR NPs or free BAR. The final concentration of BAR NPs or free BAR ranged from 0.3 to 3 μM based on the previously determined IC50 of free BAR (1.3 μM). *P. gingivalis* was incubated with BAR NPs or free BAR at 25 °C for 30 min before transferring to wells containing *S. gordonii*.

Plates containing *P. gingivalis* and *S. gordonii* were subsequently incubated for 24 h at 37 °C in anaerobic conditions [24]. The following day, the supernatant was removed and cells were washed with PBS. Adherent cells were fixed with 4% (w/v) paraformaldehyde and the cover glass was mounted on a glass slide. Biofilms were visualized using a Leica SP8 confocal microscope (Leica Microsystems Inc., Buffalo Grove, IL) under 60× magnification. Background noise was minimized using software provided with the Leica SP8 and three-dimensional z-stack biofilm images were obtained from 30 randomly chosen frames using a z-step size of 0.7 μm. Images were analyzed with Volocity image analysis software (version 6.3; Perkin Elmer, Waltham, MA, USA) to determine the ratio of green to red fluorescence (GR), representing *P. gingivalis* and *S. gordonii*, respectively. Control samples were used to subtract background levels of auto-fluorescence. Briefly, triplicate samples of *S. gordonii* alone were immobilized without *P.g* or BAR in 12-well culture plates and the same procedures for dual-species biofilm were followed. *S. gordonii*-only coverslips were visualized and images were analyzed using the previously mentioned approach. GR background was subtracted using the following formula: GR sample or control − GR *S. gordonii*-only. Each treatment group (BAR NPs or free BAR) was analyzed in triplicate and three independent frames were measured for each well. The mean and variation (SD) between samples were determined using analysis of variance (ANOVA) and differences were considered to be statistically significant when p < 0.05. The percent inhibition of *P. gingivalis* adherence was calculated with the following formula: GR sample/GR control.

### Biofilm disruption assay

The same procedures utilized in the inhibition assay were followed, except *P. gingivalis* was allowed to adhere to *streptococci* in the absence of BAR peptide or BAR NPs to demonstrate the ability of BAR-encapsulated NPs to disrupt or “treat” pre-established biofilms. The resulting *P. gingivalis/S. gordonii* biofilms were then treated for 3 h with free BAR or BAR-encapsulated NPs at various concentrations and processed and analyzed as described above.

### Inhibitory kinetics of BAR released from BAR-encapsulated NPs

Due to the similar release properties of BAR from PLGA and mPEG-PLGA NPs, PLGA NPs were selected to further assess the ability of NPs to release therapeutically relevant concentrations of BAR at different time points. PLGA BAR NPs (1.3 μM) were incubated with gentle agitation in PBS (pH 7.4) at 37 °C. After 1, 2, 4 and 8 h, the NP suspension was centrifuged, and the supernatant was collected for biofilm experiments. The NPs were re-suspended with new PBS. *P. gingivalis* was incubated with BAR NP eluate for 30 min, and subsequently transferred to a well containing an *S. gordonii* biofilm. The same biofilm inhibition assay procedure detailed above was used to visualize and analyze the samples.

### Time-dependent comparison between free BAR, BAR-encapsulated, and BAR surface-modified NPs

In addition to delivering high concentrations of BAR during the time frame of interest, the temporal evaluation of BAR activity against established biofilms was evaluated and compared. Both BAR-encapsulated and BAR surface-modified NPs were assessed due to their previously demonstrated efficacy. *P. gingivalis* was allowed to adhere to *streptococci* in the absence of peptide, then BAR (3 μM), BAR-encapsulated, and BAR surface-modified NPs (1.3 and 3 μM) were applied to the biofilms. The biofilms were assessed 1, 2, and 3 h post-administration and visualized as described above.

## Results

### Nanoparticle characterization

**Fig. 1:**
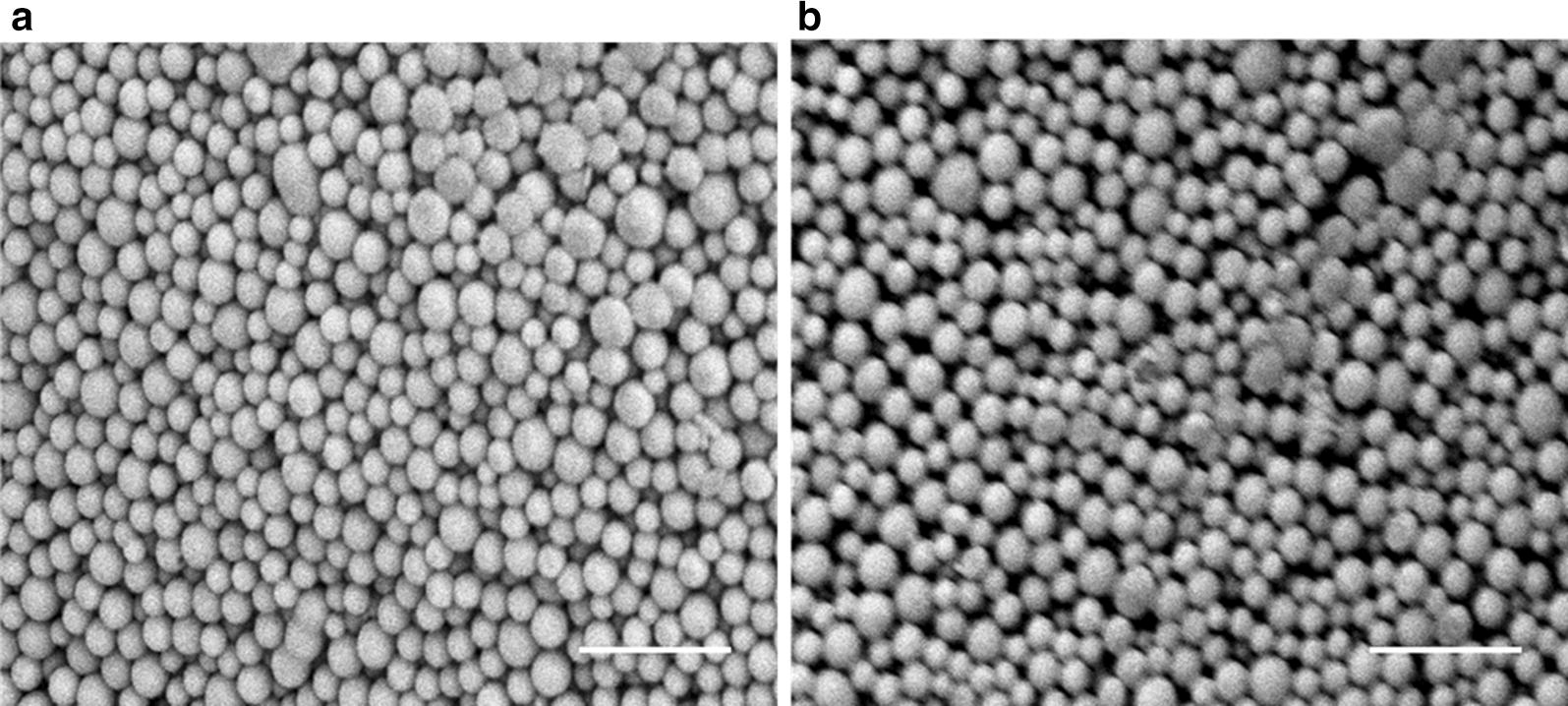
SEM images of BAR-encapsulated **a** PLGA NPs and **b** mPEG-PLGA NPs. Scale bars represent 1 μm

The morphology, size, and zeta potential of BAR PLGA and mPEG-PLGA NPs were determined. The morphologies of BAR-encapsulated PLGA and mPEG-PLGA NPs are shown in Fig. 1. Both PLGA and mPEG-PLGA NPs demonstrated spherical morphology with average unhydrated diameters of 227.5 ± 23.0 nm and 243.1 ± 31.2 nm respectively (Table 1). In comparison, the average hydrated diameters of PLGA and mPEG-PLGA NPs were 234.4 ± 19.2 nm and 278.9 ± 13.8 nm, respectively. PLGA and mPEG-PLGA NPs had zeta potentials of − 13.1 ± 0.4 mV and − 5.9 ± 0.1 mV.

**Table 1:** Physical characterization of NPs

NP type	Unhydrated diameter (nm)	Hydrated diameter (nm)	Zeta potential (mV)
PLGA NPs	227.5 ± 23.0	234.4 ± 19.2	− 13.1 ± 0.4
mPEG-PLGA NPs	243.1 ± 31.2	278.9 ± 13.8	− 5.9 ± 0.1

### Quantification of BAR loading and release

The loading of BAR peptide in PLGA and mPEG-PLGA NPs was determined using fluorescence spectroscopy, and the fluorescence was compared to a known standard of F-BAR. Loading experiments demonstrated that both PLGA and mPEG-PLGA NPs highly encapsulated BAR with 19.0 ± 0.1 and 16.1 ± 0.2 µg of BAR per mg of NP, respectively, corresponding to encapsulation efficiencies of 44 and 37% (Table 2).

**Table 2:** The amount of BAR (μg) loaded in PLGA and mPEG-PLGA NPs (mg)

NP type	BAR input (μg/mg)	BAR output (μg/mg)	Encapsulation efficiency (%)
PLGA NPs	43	19.0 ± 0.1	44.2
mPEG-PLGA NPs	43	16.1 ± 0.2	37.3

**Fig. 2:**
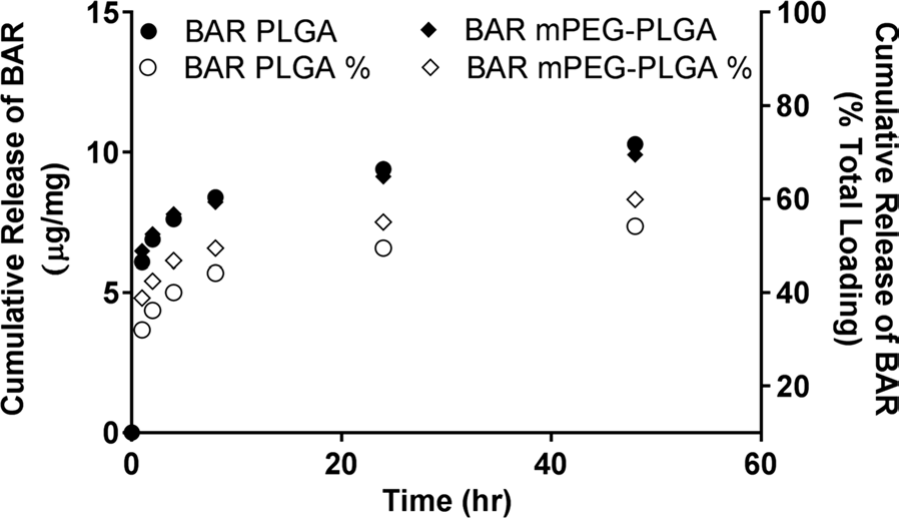
Cumulative release of BAR as a function of mass (μg BAR per mg NP, open symbols) and percent of total BAR loaded (closed symbols) over 48 h

To assess BAR release from the NPs, the fluorescence of supernatant from 1, 2, 4, 8, 24, 48 h release time points was measured and compared to a known standard of F-BAR in PBS. Release experiments demonstrated that 47% of encapsulated BAR (10.3 μg/mg) was released from PLGA NPs, while 56% of BAR (9.9 μg/mg) was released from mPEG-PLGA NPs within 24 h (Fig. 2).

### Inhibition (or prevention) of *P. gingivalis/S. gordonii* biofilm formation

**Fig. 3:**
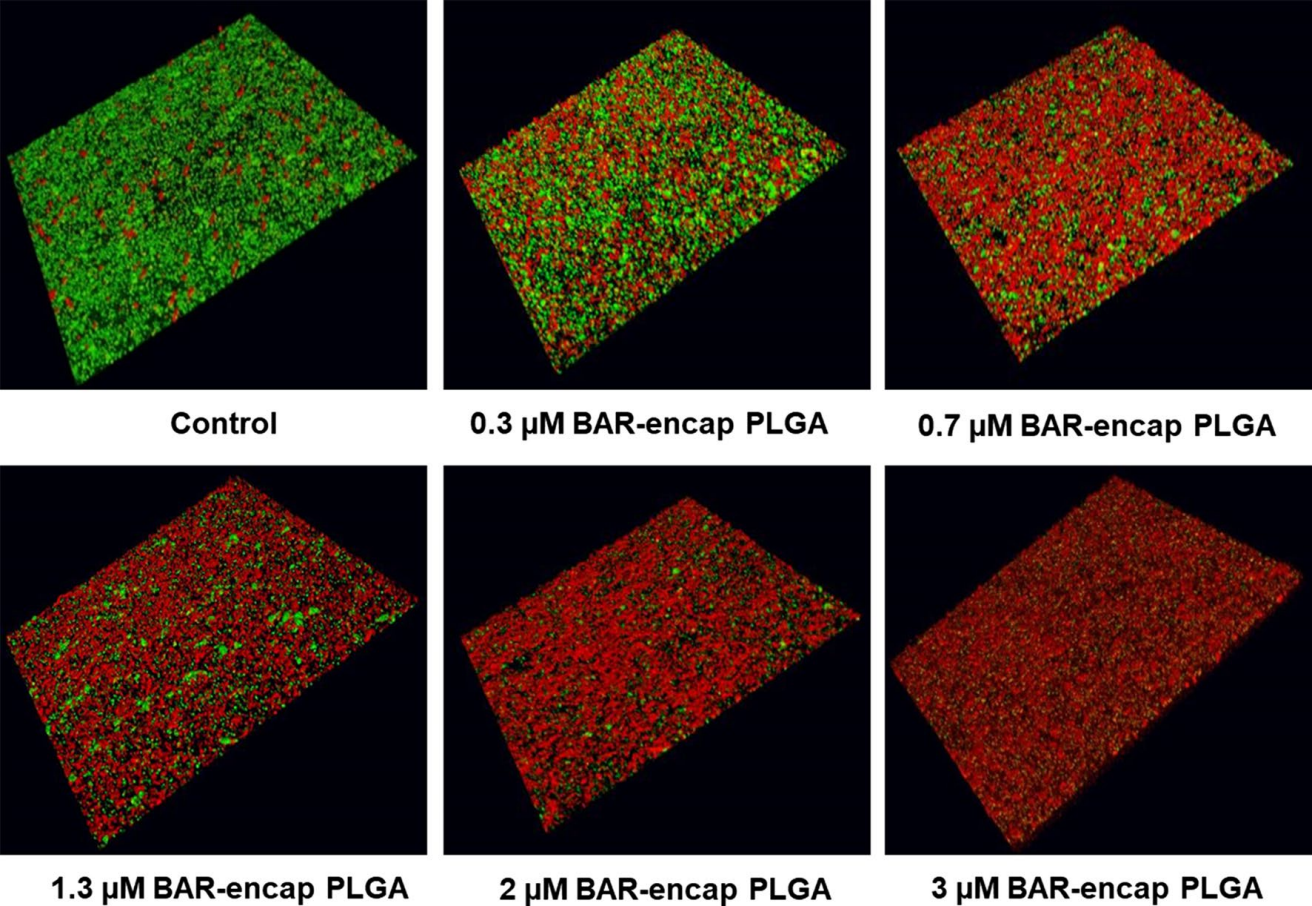
BAR-encapsulated PLGA NPs prevent *P. gingivalis* adherence to *S. gordonii*. Biofilms were visualized with confocal microscopy and the ratio of green (*P. gingivalis*) to red (*S. gordonii*) fluorescence in z-stack images was determined using Volocity image analysis software. Each grid represents 21 μm

BAR-encapsulated PLGA and mPEG-PLGA NPs were functionally evaluated to determine their potential to inhibit *P. gingivalis* adherence to *S. gordonii* after 24 h, relative to free BAR. As shown in Fig. 3 and Additional file 1, *P. gingivalis* adherence was significantly reduced in the presence of BAR-encapsulated PLGA and mPEG-PLGA NPs. Adherence was inhibited by 39% at the lowest administered concentration (0.3 μM), 59% at 0.7 μM, and reached maximum inhibition (94%) at the highest concentration of PLGA NPs tested (3 μM). Similar inhibitory results were observed for mPEG-PLGA NPs, where *P.g/S.g* biofilm formation was inhibited by 37, 55, and 92% at concentrations of 0.3 μM, 0.7 μM and 3 μM respectively. The ability of BAR-encapsulated NPs to inhibit biofilm formation was dose-dependent (IC50 = 0.7 μM) with no statistically significant differences between PGLA and mPEG-PLGA BAR-encapsulated NPs (p > 0.05). Moreover these results indicate that a lower concentration of BAR is required if incorporated within NPs, relative to free BAR administration (IC50 = 1.3 μM) (Figs. 3 and 5a).

### Disruption (or treatment) of *P. gingivalis/S. gordonii* biofilms

**Fig. 4:**
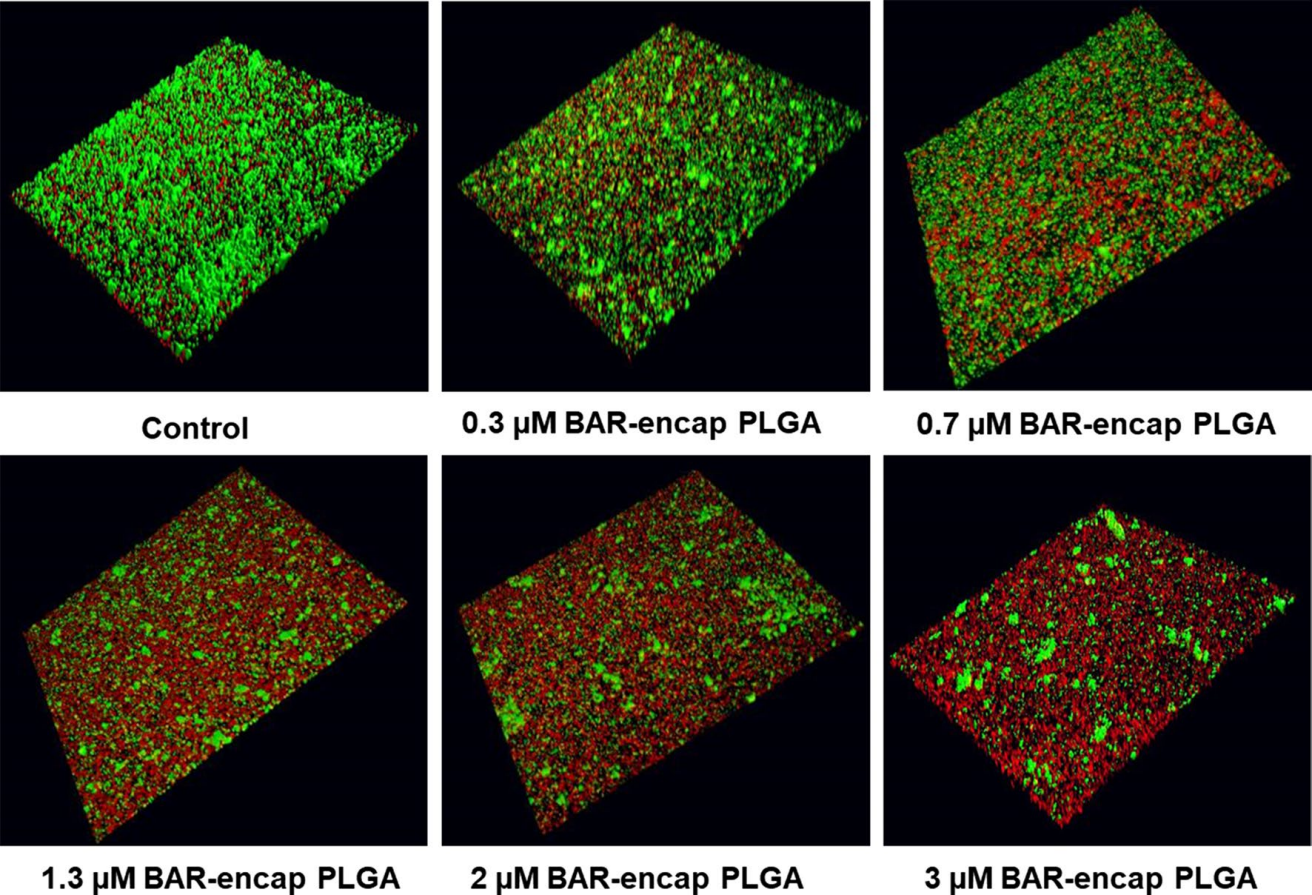
BAR-encapsulated PLGA NPs disrupt pre-established *P. gingivalis*–*S. gordonii* biofilms. Biofilms were visualized with confocal microscopy and the ratio of green (*P. gingivalis*) to red (*S. gordonii*) fluorescence in z-stack images was determined using Volocity image analysis software. Each grid represents 21 μm

**Fig. 5:**
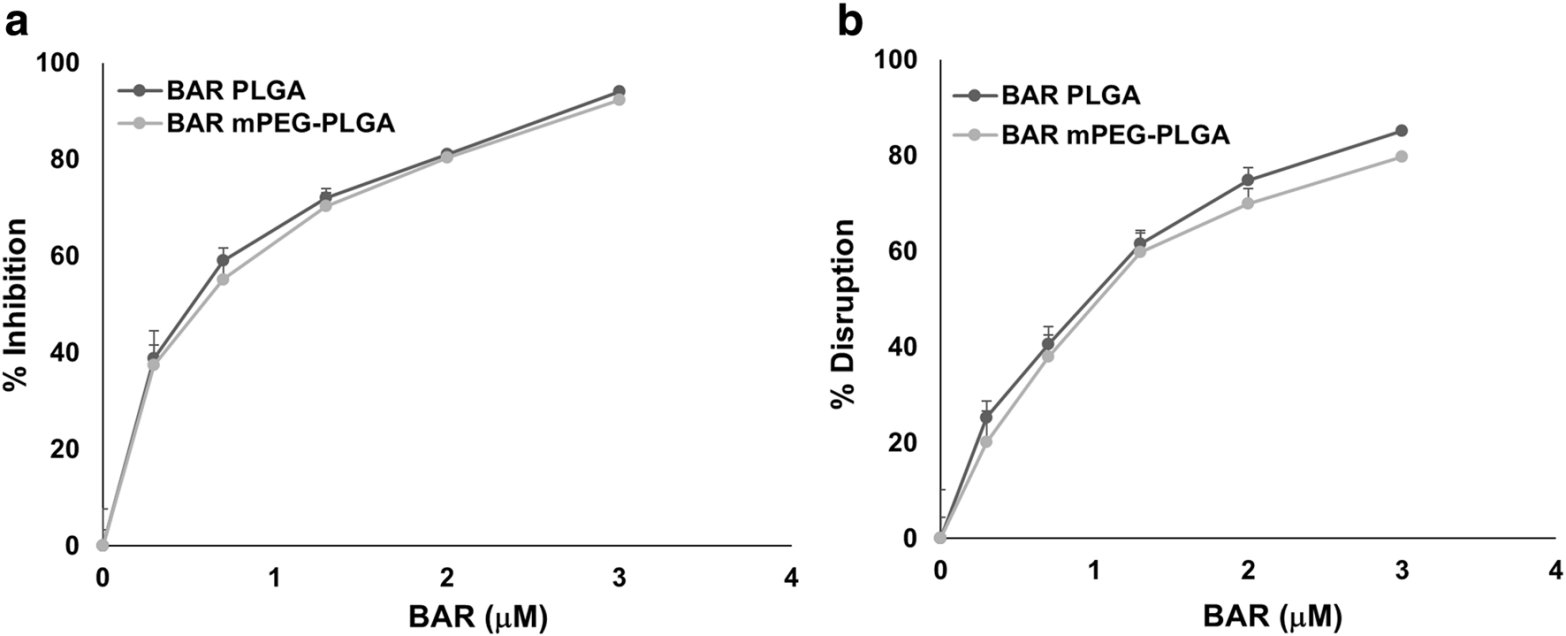
Comparison of the concentration of BAR-encapsulated PLGA and mPEG-PLGA NPs needed to **a** inhibit or **b** disrupt *P. gingivalis*/*S. gordonii* biofilms

To determine whether BAR peptide is capable of disrupting pre-existing *P. gingivalis/S. gordonii* biofilms, dual-species biofilms were formed in PBS in the absence of BAR peptide for 24 h, and were subsequently incubated for 3 h with BAR-encapsulated PLGA or mPEG-PLGA NPs. Various molar concentrations of BAR NPs ranging from 0.3 to 3 μM were tested. The biofilms were visualized and the percent inhibition was calculated as described above. As shown in Fig. 4 and Additional file 2, BAR-encapsulated PLGA and mPEG-PLGA NPs disrupted pre-existing dual-species biofilms by ~ 25% with the lowest administered concentration (0.3 μM), 40% with 0.7 μM, and 85% with 3 μM of BAR-encapsulated PLGA NPs. Similar trends were observed for the disruption of pre-existing biofilms with 0.3, 0.7, and 3 μM mPEG-PLGA NPs (20%, 38%, and 80% disrupted). Overall the IC50 values of PLGA and mPEG-PLGA (~ 1.3 μM) NPs for biofilm disruption were not statistically different (p > 0.05, Fig. 5b); demonstrating statistically significant improvements in efficacy relative to free BAR (p < 0.05).

### Inhibitory activity of BAR released from BAR-encapsulated NPs

**Fig. 6:**
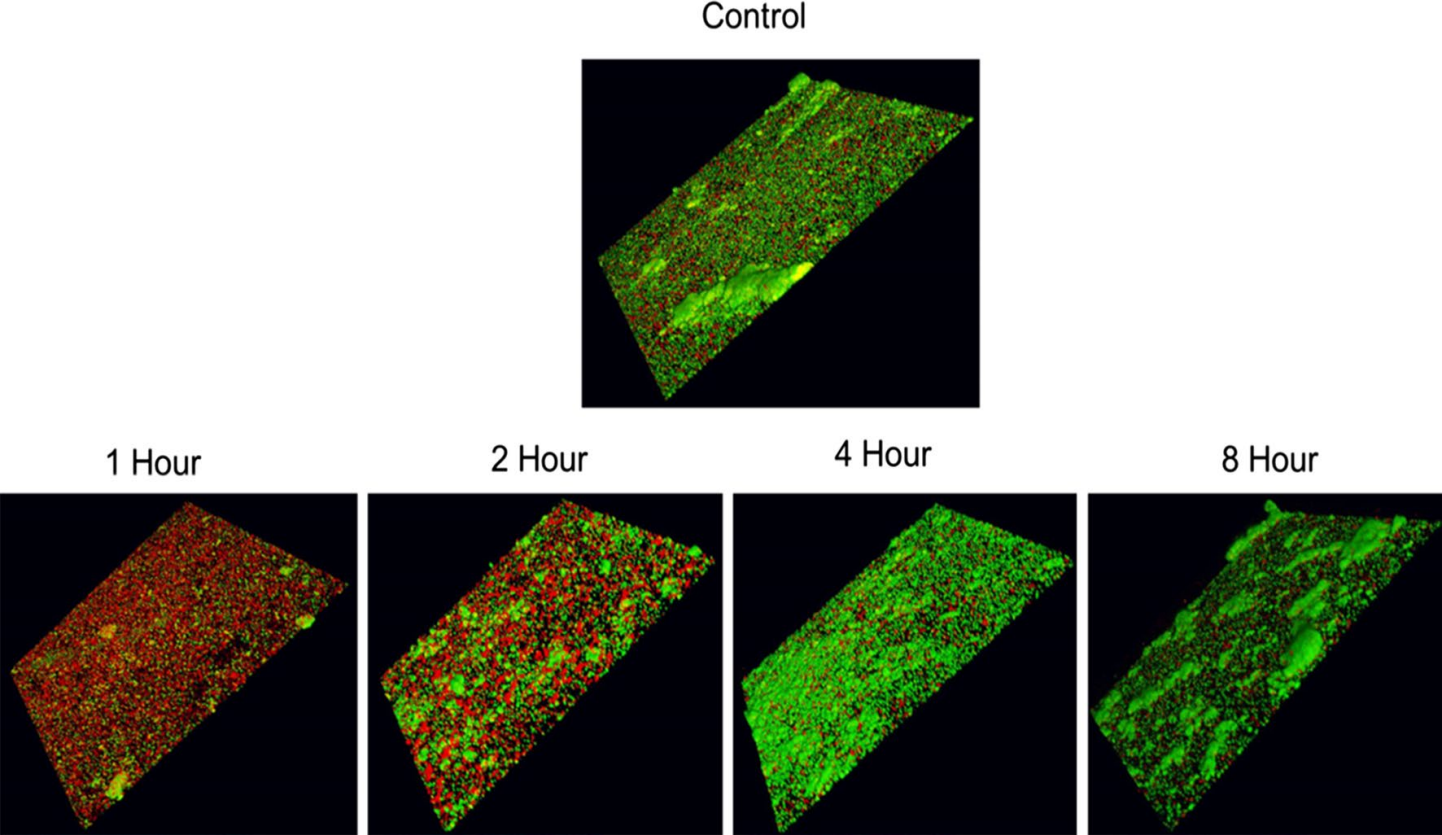
BAR-encapsulated PLGA NPs inhibit *P. gingivalis* adherence to *S. gordonii* after different durations of release. Biofilms were visualized with confocal microscopy and the ratio of green (*P. gingivalis*) to red (*S. gordonii*) fluorescence in z-stack images was determined using Volocity software. Each grid = 21 μm

To determine the inhibitory potential of BAR-encapsulated NPs, as a function of release duration, *streptococcal* cells were immobilized and *P. gingivalis* was incubated with eluate released from 1.3 μM BAR-encapsulated PLGA NPs at 1, 2, 4, and 8 h. BAR-encapsulated PLGA NPs were selected due to their similar release and inhibitory properties, relative to mPEG-PLGA NPs. As shown in Fig. 6, BAR released during the first 2 h, potently inhibited biofilm formation (68% and 32%, respectively), whereas BAR released after 4 and 8 h provided less potent inhibition of biofilm formation (25% for both time points). These results indicate that BAR-encapsulated NPs release an inhibitory dose of peptide for at least 2 h.

### Time-dependent comparison of free BAR, BAR-encapsulated, and BAR surface-modified NP biofilm disruption

**Fig. 7:**
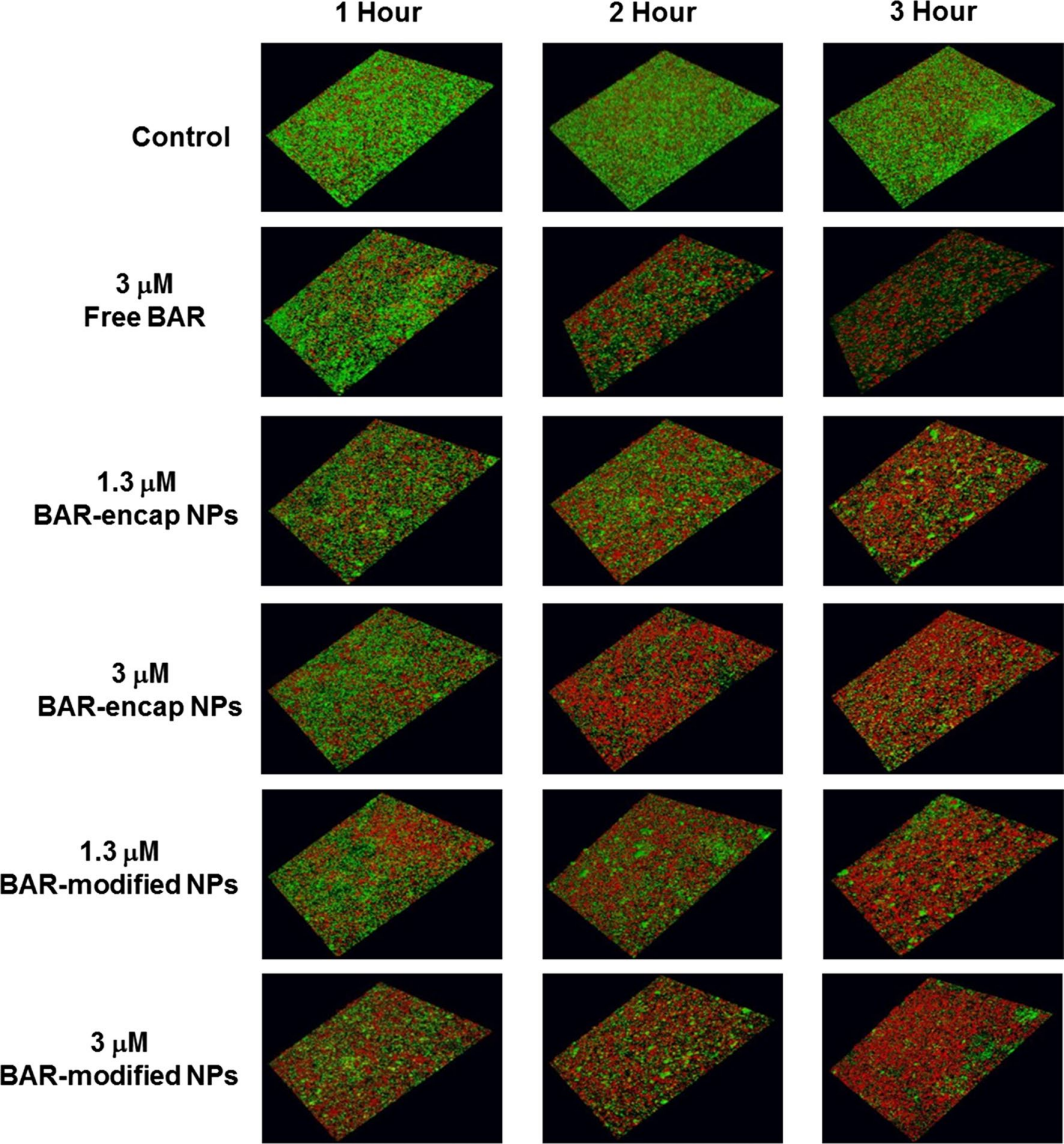
Disruption of established *P. gingivalis/S. gordonii* biofilms after different exposure times to BAR surface-modified NPs, BAR-encapsulated NPs and free BAR. Biofilms were visualized with confocal microscopy and the ratio of green (*P. gingivalis*) to red (*S. gordonii*) fluorescence in z-stack images was determined using Volocity software. Each grid = 21 μm

Previous studies demonstrated that BAR surface-modified PLGA NPs potently disrupt pre-established *P. gingivalis/S. gordonii* biofilms [24]. To compare the temporal effect resulting from the administration of the newly formulated BAR-encapsulated NPs, relative to free BAR or previously tested BAR surface-modified NPs, two concentrations of BAR-encapsulated and BAR surface-modified PLGA NPs were compared with free BAR after 1, 2, and 3 h administration to pre-established biofilms. As shown in Table 3 and Fig. 7, free BAR (3 μM) minimally disrupted pre-existing biofilms during the first hour of application (23%), and demonstrated a slight increase in disruption after 2 h (44%). After 3 h, free BAR (3 μM) disrupted 69% of the pre-existing biofilm. In comparison, administration of the same equimolar concentration of BAR-encapsulated NPs (1.3 and 3 μM) disrupted the established biofilm during the first hour of exposure by 32% and 38%, respectively and demonstrated even more potent disruption (47% and 52%) after 2 h. The maximum disruption for 1.3 and 3 μM doses (66% and 77%, respectively) was achieved after 3 h exposure to biofilms. Comparatively, both 1.3 and 3 μM BAR surface-modified NPs disrupted pre-existing biofilms within 1 h by 43% and 49%, respectively, and induced more potent biofilm disruption (59% and 69%) after 2 h exposure, demonstrating statistically significant disruption, relative to disruption induced by free BAR peptide. The highest levels of disruption (71% and 83% respectively) were achieved after 3 h BAR surface-modified NP administration. Overall, BAR surface-modified NPs were statistically more effective than free BAR (p < 0.05) in disrupting established biofilms after 1, 2, and 3 h administration. However, no statistical differences were observed for BAR-encapsulated NPs (p > 0.05), relative to BAR surface-modified NPs or free BAR peptide after 1, 2, or 3 h administrations.

**Table 3:** Percent disruption of pre-existing biofilms with different treatment groups and concentrations

Time (h)	% Disruption of pre-formed biofilms				
	Free BAR (3 μM)	BAR-mod NPs (1.3 μM)	BAR-mod NPs (3 μM)	BAR-encap NPs (1.3 μM)	BAR-encap NPs (3 μM)
1	22.6 ± 0.2	43.4 ± 0.2	48.9 ± 0.1	32.3 ± 0.1	37.7 ± 0.1
2	44.4 ± 0.2	59.2 ± 0.1	68.7 ± 0.1	46.6 ± 0.2	52.4 ± 0.2
3	69.0 ± 0.0	71.2 ± 0.1	83.4 ± 0.0	66.1 ± 0.1	77.0 ± 0.0

## Discussion

*Porphyromonas gingivalis* has been identified as a “keystone” pathogen involved in the initiation and progression of periodontal inflammatory disease, by disrupting host-microbe homeostasis and inducing population changes in the subgingival biofilm. This disruption and colonization is initially prompted by the association of *P. gingivalis* with oral *streptococc*i in the supragingival niche, and is thus an ideal target for therapeutic intervention [5]. Previous studies have shown that BAR peptide inhibits biofilm formation by *P. gingivalis* and *S. gordonii* in vitro and reduces the virulence of *P. gingivalis* in a murine model of infection [15–17]. While efficacious, BAR effectiveness was limited by the duration of exposure within the oral cavity, and necessitated a higher concentration to disrupt previously established biofilms [15–17]. In previous work we sought to address these challenges by synthesizing BAR surface-modified NPs to multivalently inhibit biofilm formation [24]. The goal of this study was to develop, characterize, and compare BAR-encapsulated NPs that release BAR within a time frame relevant to delivery in the oral cavity.

Nanoparticle characterization revealed that PLGA and mPEG-PLGA BAR-encapsulated NPs exhibited spherical morphologies and average particle diameters of 234.4 ± 19.2 nm and 278.9 ± 13.8 nm, with respective zeta potentials of − 13.1 ± 0.4 mV and − 5.9 ± 0.1 mV. These values are in agreement with expected values for these polymeric NPs [24–26, 28, 29]. Both PLGA and mPEG-PLGA NPs were synthesized with 43 μg of BAR per mg NP, corresponding to loading concentrations deemed feasible for biofilm inhibition with free BAR [15–17]. PLGA and mPEG-PLGA NPs demonstrated relatively high peptide loading with 19.0 ± 0.1 and 16.1 ± 0.2 µg BAR per mg of NP respectively.

In addition to high loading, PLGA and mPEG-PLGA NPs released 40% and 48% of BAR within the first 4 h, with no statistically significant differences between release profiles. The NP formulations were designed to achieve therapeutic concentrations of BAR in the oral cavity for a minimum of 2 h. This initial window of 2 h release was targeted as we envision formulating NPs in a mouth rinse or toothpaste product. Ideally, in future formulations, we seek to tailor the release of peptide for up to 12 h since we envision these formulations may be applied once or twice daily, to exert immediate effect over a number of hours.

To assess the functionality of BAR-NPs, the inhibition and disruption concentrations of BAR-encapsulated PLGA and mPEG-PLGA NPs were determined against dual-species biofilms. As shown in Figs. 3 and 4, BAR NPs demonstrated potent inhibition and disruption with IC50 s = 0.7 μM and 1.3 μM, respectively, with negligible differences observed between PLGA and mPEG-PLGA NPs (Additional file 1 and Additional file 2). To explore the temporal effect of BAR released from PLGA NPs on biofilm inhibition (prevention) in greater depth, the efficacy of BAR-encapsulated NPs was assessed in a dual-species biofilm after 1, 2, 4 and 8 h post-application. Sufficient BAR release was achieved, relating to inhibitory concentrations of 1.3 μM during the first 4 h of administration (Fig. 6). Moreover, the temporal dependence of free BAR, BAR-encapsulated, and BAR surface-modified NPs to disrupt pre-established biofilms (treatment) was measured after 1, 2, and 3 h application. As shown in Fig. 7 and Table 3, BAR-encapsulated and BAR surface-modified NPs achieved moderate biofilm inhibition within 1 h in a dose-dependent manner; however, similar concentrations of free BAR required prolonged exposure of up to 3 h to achieve more potent effect. These results demonstrate that BAR-encapsulated NPs provide a feasible alternative to free BAR and BAR surface-modified NPs to target dual-species oral biofilms and provide rapid onset of action. Together, these studies indicate that BAR-encapsulated NPs may serve as a short-term delivery formulation to enhance BAR delivery and potency in the oral cavity. Moreover, by encapsulating versus surface-modifying NPs with BAR, these NPs may offer the potential to specifically target NPs with modifications that can complement BAR activity to engage with these or other bacterial species in future work.

To date, a variety of polymeric nanoparticle formulations have been developed for oral delivery; however, these vehicles have primarily focused on the delivery of non-specific active agents such as antibiotics [30–39]. Antibiotics such as chlorhexidine [30, 31], minocycline [32, 33], clarithromycin [36], vancomycin [34], doxycycline [37], and tetracycline [35, 38, 39] are among the antibiotics that have been incorporated into a variety of polymeric vehicles [30–37] to provide sustained-delivery, prolong activity, exert antibacterial activity, and decrease antibiotic cytotoxicity [30–37]. Yet, despite antibiotic choice, primary concerns of antibacterial resistance and cytotoxicity remain [30, 31, 35]. While chitosan and PLGA NPs that encapsulated chlorhexidine dihydrochloride (CHX) demonstrated strong adherence to tooth surfaces and sustained-release for 48 h in neutral pH conditions, moderate cytotoxicity due to CHX was observed in human gingival fibroblasts [31]. Similar studies seeking to ameliorate periodontal infection caused by *A. actinomycetemcomitans* and *P. nigrescens* with PLGA lovastatin-chitosan-tetracycline NPs demonstrated potent inhibition up to 1 week after administration. However, significantly elevated alkaline phosphatase was observed in cells treated with 0.1% or 0.3% tetracycline-loaded nanoparticles on days 7 and 9 [35]. Overall, these studies have shown that delivery vehicles have the potential to increase antibiotic effectiveness by decreasing the concentration required. However, bacterial resistance, non-specific targeting, and cytotoxicity concerns with chronic use suggest that the development of more specifically acting active agents will offer safer alternatives for biofilm inhibition.

More recently, specifically targeted biological agents have been investigated to treat periodontal diseases. Delivery of *thyA* gene [40], Punica granatum extract [41], *H. madagascariensis* leaf extract [42], miR-146a [43], and the anti-inflammatory agent 15d-PGJ2 [44] have been investigated to vaccinate against and target periodontal diseases. Recent work assessed the delivery of an oral vaccine comprised of an auxotrophic complementation of the *thyA* gene to produce an immune response against *S. gordonii.* Although this study demonstrated promise utilizing *S. gordonii* as a live oral vaccine, to date there are few formulations available to localize or sustain biologic administration to the oral cavity [40]. In other work, PLGA NPs encapsulating a novel anti-inflammatory agent (15d-PGJ2), demonstrated promise in reducing inflammatory response and bone resorption in mouse model of periodontitis after daily administration [44], demonstrating the feasibility of combined biologic and delivery vehicle against oral pathogens. Despite this recent progress in the delivery of biological agents for oral applications, currently few biological agents in combination with delivery vehicles have been developed to inhibit keystone-specific interactions during the initial stages of periodontal disease [24].

In addition to progress in the development of vehicles to encapsulate antibiotic and biological agents in polymeric delivery vehicles, polymeric platforms have also been surface-modified with a variety of molecules including RGD [33], chitosan [31, 36], tertiary amines bearing two t-cinnamaldehyde substituents [45], dimethyl-octyl ammonium [45], and BAR peptide [24] to increase the mucoadhesivity (and in the latter case, specificity) of oral delivery formulations. A variety of polymers have been modified with biological ligands to impart enhanced therapeutic effect [24, 33]. As one example, the delivery of antibiotic minocycline-loaded poly(ethylene glycol)–poly(lactic acid) (PEG–PLA) nanoparticles have targeted oral epithelial cells by surface-modification with RGD peptides. Surface-modification of PEG–PLA NPs increased epithelial cell attachment and maintained effective drug concentrations in gingival fluid for more than 2 weeks in vivo, relative to unmodified minocycline NPs. Similarly, chitosan-modified polyvinyl caprolactam-polyvinyl acetate-polyethylene glycol graft copolymer (Soluplus) and poly-(dl-lactide-*co*-glycolide) nanoparticles loaded with clarithromycin, increased antibacterial efficacy and provided sustained-release against oral biofilms [36]. Although this study demonstrated effective treatment of periodontitis, the limitations of antibiotic delivery still pose challenges [33]. Surface modification of nanoparticles has imparted new attributes to target active agents to oral-specific niches. We expect that combining our current work, with surface functionalization demonstrated in our previous study [24], may confer additional advantages in targeting keystone species by providing prevention and treatment via adhesion and a localized release-mediated platform.

Taken together, our results demonstrate that BAR-encapsulated NPs achieve more potent in inhibition and disruption than equimolar free BAR administration. We believe that incorporation of BAR peptide in NPs provides gradual release of BAR peptide, while BAR-modification offers a platform to provide a higher localized concentration of BAR in the oral cavity via multivalent interactions. BAR-encapsulated NPs offer a platform to improve efficacy, and potentially longevity in the oral cavity compared to the transient activity of free BAR. These experimental results will be helpful in developing NPs in therapeutic formulations such as toothpaste, mouth rinse or chewing gum. Future studies may focus on developing blended polymeric NPs to more gradually release inhibitory concentrations for 8–12 h. Moreover, combining this platform with surface functionality to provide mucoadhesive or specific interactions with gingival tissue may be pursued to enhance the targeting potential. Ongoing and future work in our groups seeks to assess the efficacy of both BAR-modified and BAR-encapsulated NPs in a murine model of periodontitis.

## Additional files

**Additional file 1.** BAR-encapsulated mPEG-PLGA NPs prevent *P. gingivalis* adherence to *S. gordonii*. Biofilms were visualized with confocal microscopy and the ratio of green (*P. gingivalis*) to red (*S. gordonii*) fluorescence in z-stack images was determined using Volocity image analysis software. Each grid = 21 μm.

**Additional file 2.** BAR-encapsulated mPEG-PLGA NPs disrupt pre-established *P. gingivalis*–*S. gordonii* biofilms. Biofilms were visualized with confocal microscopy and the ratio of green (*P. gingivalis*) to red (*S. gordonii*) fluorescence in z-stack images was determined using Volocity image analysis software. Each grid = 21 μm.
