# Supplementary figures and images for: BAR-encapsulated nanoparticles for the inhibition and disruption of Porphyromonas gingivalis–Streptococcus gordonii biofilms
Source: J Nanobiotechnology. 2018 Sep 15;16:69. doi: 10.1186/s12951-018-0396-4 (PMC6138925; doi:10.1186/s12951-018-0396-4)

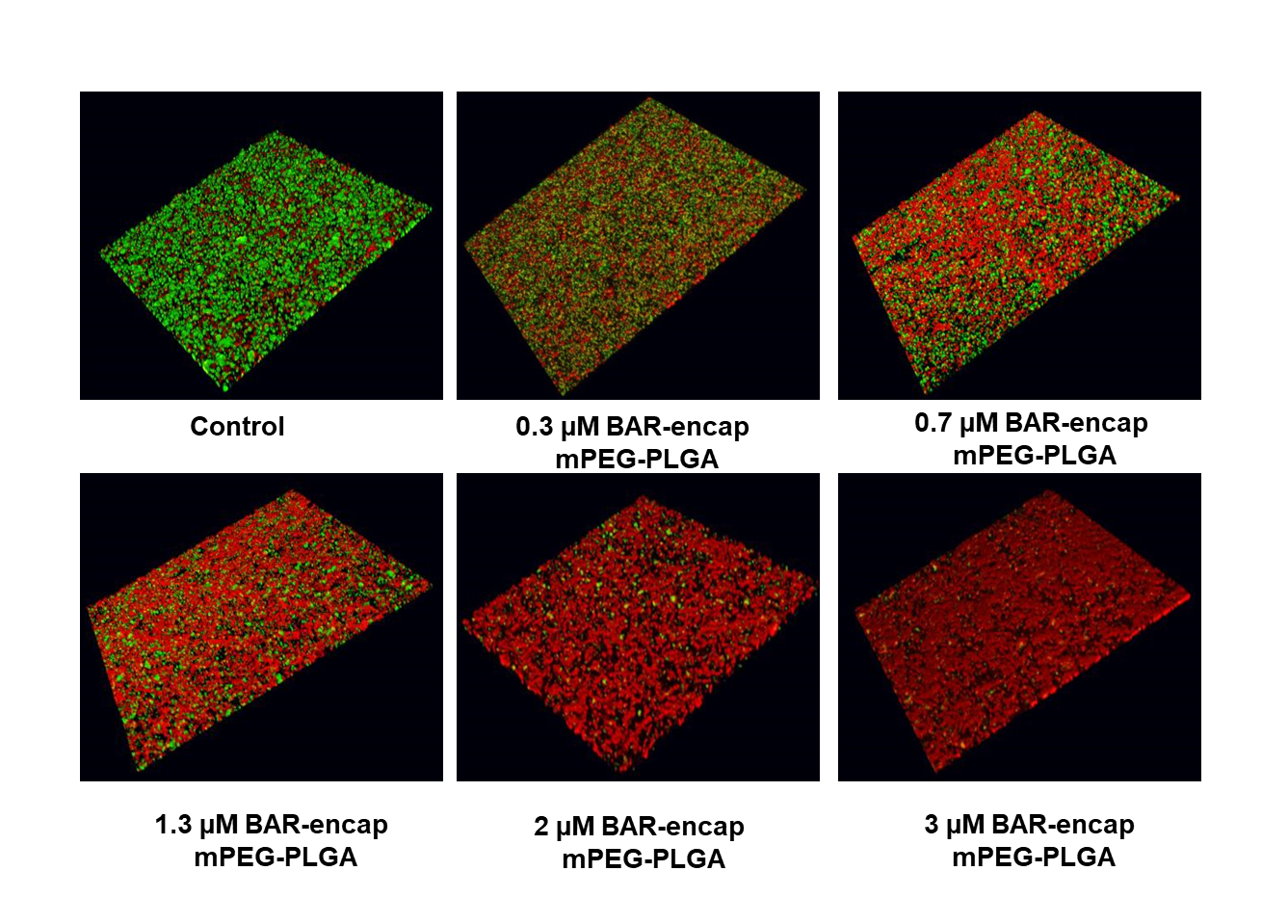

Supplement: Supplementary file 1 — Additional file 1. BAR-encapsulated mPEG-PLGA NPs prevent P. gingivalis adherence to S. gordonii. Biofilms were visualized with confocal microscopy and the ratio of green (P. gingivalis) to red (S. gordonii) fluorescence in z-stack images was determined using Volocity image analysis software. Each grid = 21 μm. [file 12951_2018_396_MOESM1_ESM.tif]

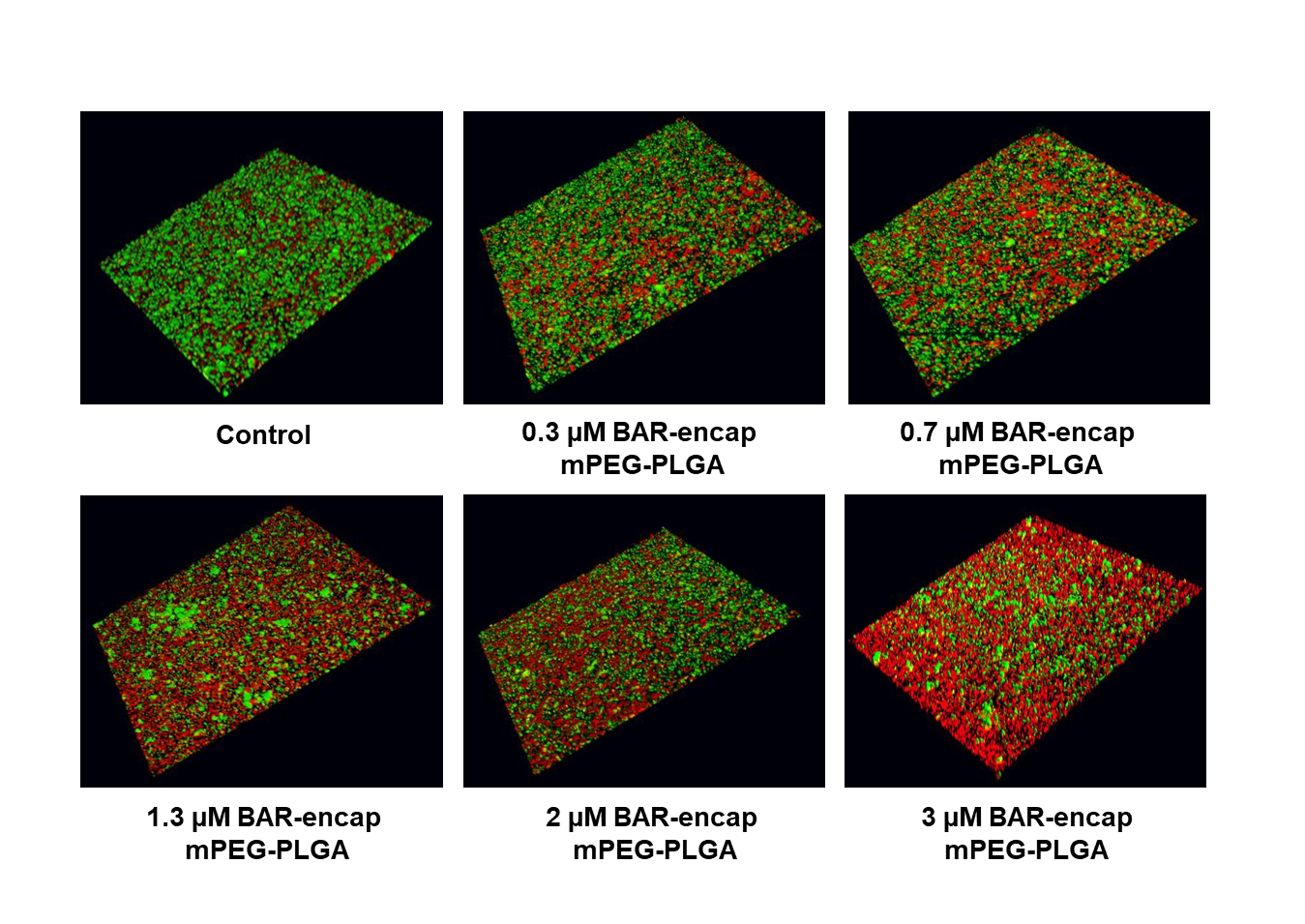

Supplement: Supplementary file 2 — Additional file 2. BAR-encapsulated mPEG-PLGA NPs disrupt pre-established P. gingivalis–S. gordonii biofilms. Biofilms were visualized with confocal microscopy and the ratio of green (P. gingivalis) to red (S. gordonii) fluorescence in z-stack images was determined using Volocity image analysis software. Each grid = 21 μm. [file 12951_2018_396_MOESM2_ESM.tif]
